# Facile surface functional polyetheretherketone with antibacterial and immunoregulatory activities for enhanced regeneration toward bacterium-infected bone destruction

**DOI:** 10.1080/10717544.2021.1960924

**Published:** 2021-08-02

**Authors:** An’an Sun, Xi Lin, Zhiqiang Xue, Jiyue Huang, Xinxin Bai, Lingling Huang, Xinhua Lin, Shaohuang Weng, Min Chen

**Affiliations:** a Department of Orthopedic Surgery, Fujian Medical University Union Hospital, Fuzhou, China; b Department of Emergency Surgery, Center for Trauma Medicine, The First Affiliated Hospital of Fujian Medical University, Fuzhou, China; c Department of Pharmaceutical Analysis, School of Pharmacy, Higher Educational Key Laboratory for Nano Biomedical Technology of Fujian Province, Fujian Medical University, Fuzhou, China; d Department of Stomatology, The First Affiliated Hospital of Fujian Medical University, Fuzhou, China

**Keywords:** Co-deposition of polydopamine and gentamicin, PEEK, anti-infection, immunoregulatory, enhanced osseintegration

## Abstract

Existing biologically inert or unmodified implants to treat infectious bone defects or osteomyelitis still cannot effectively solve bacterial infection and osseointegration. In this work, a simple co-deposition strategy was developed to modify porous polyetheretherketone (PEEK) with improved antibacterial activity and controllable immunoregulatory ability. After PEEK was treated by H_2_SO_4_ to obtain porous PEEK (SPEEK), the self-polymerization of dopamine was operated on SPEEK in the solution of dopamine and gentamicin sulfate (GS) to prepare polydopamine (pDA) and GS layer-modified SPEEK (labeled as SPEEK–pDA–GS). The morphology, surface property, and molecular structure of SPEEK–pDA–GS were investigated. Besides the antibacterial property of SPEEK–pDA–GS ascribed to the successful immobilization of GS, SPEEK–pDA–GS exhibited promoted osseointegration through the results of mineralization, alkaline phosphatase (ALP) levels and osteogenic gene expression. Furthermore, the evaluation of the cell proliferation suggested that SPEEK–pDA–GS possessed the biocompatibility and the immunoregulatory ability that induced macrophages to anti-inflammatory M2 phenotype. Using rat as model, *in vivo* results containing X-ray, μ-CT, immunohistochemistry, and pathological analysis showed the excellent healing effect of SPEEK–pDA–GS on bone defect with infection with biological safety. This work illustrates a new insight into the simple and effective modification of PEEK and other implants with antibacterial, immunoregulatory, and osseointegration abilities for clinical requirement.

## 1. Introduction

Infected bone destruction, including infected bone defect and osteomyelitis, is difficult to cure in clinic. The combined application of bone graft and antibiotic is the common treatment. In recent years, the development of immunology and implanting modification has attracted increased attentions in the infected bone remolding (Liu et al., 2018; 2019; Xu et al., 2019; Gao et al., 2020) to achieve enhanced treated effect. Bacterial reproduction, inflammation, and immunosuppression may coexist in infected bone destruction (Ram & Chinen, 2011; Liu et al., 2019), which requires that the implantation site contains one or more functions of antibacterial activity, immunomodulatory ability, and guiding cell fate to promote osseointegration between bone and implant.

The composition and various properties of polyetheretherketone (PEEK) reveal that PEEK is a promising and excellent candidate in orthopedic/dental implants for bone healing (Adler et al., 2019; Hevesi et al., 2019; Mishra and Chowdhary, 2019; Zhang et al., 2019). Compared with metallic implants, PEEK exhibits excellent mechanical properties and good chemical resistance (Li et al., 2020; Prakash et al., 2020; Wu et al., 2020). Regrettably, the poor bioactivity of PEEK can impede integration with bone tissues ascribed to the following two main conditions. One is the presence of bacterial infections in the implantation area. The other is that the application of PEEK implant may cause serious foreign body immunereactions, like impaired immune function (Trindade et al., 2016; Walsh et al., 2018; Chandorkar et al., 2019). Therefore, PEEK implants should be modified to achieve the functions of antibacterial activity and/or immunoregulatory ability to promote osseointegration (Deng et al., 2018; 2020; Zhang et al., 2021). For example, graphene oxide and adiponectin functioned sulfonated PEEK promote infective bone healing through the combined effect of osteogenicity and photodisinfection (Deng et al., 2020). Moreover, zinc-modified sulfonated PEEK exhibits the property of bacteriostasis and immunomodulation (Liu et al., 2018), and the dexamethasone plus minocycline-loaded liposome-modified PEEK reveals antibacterial ability and anti-inflammatory property (Xu et al., 2019). These reports illustrate the feasibility of the achievement of dual- or triple-functional PEEK (Gan et al., 2016; Hong et al., 2018). It still requires a simple and cost-effective strategy without potential biotoxicity or the need of complex device for PEEK modification with promoted bioactivity (Zheng et al., 2019).

The topography and roughness of the implant surface play a critical role in cell fate and immunomodulation (Ouyang et al., 2016; Lee et al., 2019; Wei et al., 2019; Vassey et al., 2020; Xia et al., 2020). Porous PEEK can induce macrophages to the anti-inflammatory M2 phenotype (Liu et al., 2019; Wei et al., 2019), and this immunomodulation facilitates the osteogenic differentiation for the enhancement of bone healing (Bai et al., 2020; Gao et al., 2020). Moreover, bacterial infections can cause the serious consequences of implant loosening and even detachment (Busscher et al., 2012; Campoccia et al., 2013; Braem et al., 2014; Yan et al., 2018; Xue et al., 2020). Therefore, aside from immunomodulation, the PEEK implant also needs improved antibacterial ability to combat potential infection and reduce the risk of implant loosening especially in surgical areas trapped by bacterial infections. The use of copper nanoparticles (Liu et al., 2019), zinc layer (Liu et al., 2018), or silver particles (Yan et al., 2018) or the composite of CuO/Ag (Yan et al., 2020) on porous PEEK is fabricated to improve the antibacterial ability of implant. However, the potential toxicological effects of metal ions may bring serious damage (Schrand et al., 2010; Makhdoumi et al., 2020), which limits the application prospects. The development of convenient procedure to improve the surface of PEEK and realize the multifunction of PEEK for the promotion of bone integration remains a concern. Ascribed to the broad spectrum antibacterial activity and good stability, gentamicin (GS) is especially used for the treatment of implant-related infections and local bone infection (Chang et al., 2006; Javier et al., 2019). In addition, several antibacterial therapies using biopolymeric, polymeric scaffolds (Lemnaru (Popa) et al., 2020; Zarayneh et al., 2018; Lu et al., 2018) and other biological antibacterial strategies (Sunderland et al., 2017; Jasim et al., 2018; Shahraki & Mirsaeidi, 2021) have been developed. Compared with the reported mediums for the fabrication of antibacterial system, polydopamine (pDA), which can self-polymerize on the variable solid surface under suitable conditions (Weng et al., 2015; Ryu et al., 2018), has emerged as an effective tool for biomolecular immobilization (Bai et al., 2020; Fu et al., 2021), metal deposition (Li et al., 2020), and metal oxide entrapment (Xian et al., 2020) for biomedical applications including disinfection due to integrated activity. However, most of the applications of pDA coating are based on the application of residual –NH_2_ or –OH for the covalent conjugate (Kwon & Bettinger, 2018; Qi et al., 2019; Jin et al., 2020). The direct simple immobilization of active molecules (like antibiotic) on the pDA layer in the assembly of pDA on the solid surface to obtain the biofunctional activity is rare.

Our recent work illustrated that the PEEK modification was achieved using the immobilization of antibiotic through the *in situ* construction of brushite to effectively promote osseointegration in the infected condition (Xue et al., 2020). Together with the potential deposition ability of pDA, we assume that the self-polymerization and the assembly process of pDA on PEEK will induce some drug co-deposition on the solid surface. First, porous PEEK (SPEEK) with the removal of sulfur was fabricated. Then, dopamine was self-polymerized and deposited on SPEEK to be pDA, which acted as a bridge for the co-deposition of GS to form SPEEK–pDA–GS. The fabricated procedure induced the dual function of antibacterial property and controlled immunoregulation of macrophage to the anti-inflammatory M2 phenotype. The proposed design is to achieve the rapid sterilization of PEEK implants through the attachment of antibiotic in the early stage of acute inflammation and control infection in case of bacterial infection. The immunoregulatory ability of the SPEEK–pDA–GS promotes the development of inflammation in a direction conducive to tissue repair to treat the bone defect accompanied by infection.

## 2. Materials and methods

### 2.1. Sample preparation and modification

Medical grade PEEK samples with different sizes (cylindrical samples: Φ 4 mm × 2 mm; Φ 10 mm × 2 mm; Φ 10 mm × 10 mm, and Φ 2 mm × 5 mm, Φ = diameter) were tailor-made and obtained for this work. All PEEK samples were cleaned using acetone, ethanol and then deionized water. Simple PEEK sample was immersed in concentrated sulfuric acid (95–98%) for 3 min. Then, the sample was washed thoroughly with deionized water. After that, the residual sulfur was removed by hydrothermal treatment at 120 °C for 4 h, such treated PEEK called as SPEEK. Dopamine hydrochloride (Sigma-Aldrich, USA) and gentamicin sulfate (Sigma-Aldrich, USA) were diluted in Tris-HCl solution (10 mM, pH 8.5) to be a final concentration of 2 mg/ml and 3 mg/ml, respectively. Then, SPEEK was immersed in the above solution in the dark for 12 h reaction. Then, the product was rinsed with sterile water to wash off the unabsorbed product or reactant. The obtained sample was named as SPEEK-pDA-GS. While, SPEEK-pDA was prepared from the same procedure of SPEEK-pDA-GS in the solution without GS.

### 2.2. Mechanical testing

The compression property of the different PEEK samples (Φ 10 mm × 10 mm) was carried out with AGS-X universal material testing machine (Shimadzu, Japan). Using compression mode, all of the samples were tested at a speed of 1 mm/min with the max capacity of 8000 Newton in 3 times. And the results were read from the corresponding analysis software.

### 2.3. Release of GS in SPEEK-pDA-GS in simulated body fluid (SBF)

SPEEK-pDA-GS prepared from PEEK (Φ 4 mm × 2 mm) was immersed in 500 μL SBF and cultured at 37 °C for the GS release evaluation. Using O-phthalaldehyde (OPA) derivatization (Gubernator et al., 2006), the 500 μL solution was extracted for the determination of GS at different times with the correspondingly supplemented 500 μL SBF at each extraction. Excited at 340 nm, the emission fluorescence of the OPA derivatized GS was tested by Cary Eclipse fluorescence spectrophotometer (Agilent Technologies, USA). Finally, the total release of GS from SPEEK-PDA-GS was calculated.

### 2.4. Antibacterial test

*Staphylococcus aureus* (*S. aureus*, ATCC 6538) and *Escherichia coli* (*E. coli*, ATCC 25922) were applied to evaluate the antimicrobial activities of the same shape of PEEK (Φ 4 mm × 2 mm), SPEEK, SPEEK-pGA and SPEEK-pDA-GS according to the reported procedure with a minor revision (Liu et al., 2019; Xue et al., 2020). A 50 μL of bacterial suspension (∼1.0 × 10^7^ cfu/mL) was dropped onto the respective sample. After incubation at 37 °C for 24 h, the samples with bacterium were respectively shaken in saline for 1 min to dissociate the bacteria. After that, 100-fold diluted bacterial suspension was cultured in Luriae-Bertani (LB) medium at 37 °C for 16–18 h. The bacterial colonies of different groups were quantified and compared. The antimicrobial rate was counted using the equation of (C − T)/C × 100%. The C and T are the respective bacterial colony (cfu/sample) on PEEK and modified samples (SPEEK, SPEEK-pDA and SPEEK-pDA-GS).

### 2.5. The evaluation of cell proliferation and cell viability using CCK-8

The cell proliferation and viability on different PEEK samples (Φ 10 mm × 2 mm) were evaluated using CCK-8 method (Dojindo, Japan) according to the testing instructions.

### 2.6. Osteo-differentiation

The alkaline phosphatase (ALP) activity of MC3T3-E1 cells was monitored using ALP kit (Beyotime Biotechnology, China). In a 24-well plate, 3 × 10^4^ MC3T3-E1 cells were respectively seeded in a plate with the placed implant sample. After a certain period of incubation, the ALP activities of MC3T3-E1 cells at different PEEK (Φ 10 mm × 2 mm) samples were tested with the manufacturer's instructions.

Place the sample in a 24-well plate in advance, and seed each well with 2 × 10^3^ MC3T3-E1 cells in a plate with the placed PEEK (Φ 10 mm × 2 mm) samples, and incubate at 37 °C for 14 days and 21 days to evaluate the mineralization of the extracellular matrix. The surface of the natural PEEK or modified PEEK samples was gently washed with 3 times using PBS, and fixed with 4% paraformaldehyde at 37 °C. Then, the sample was immersed in the alizarin red S (ARS, Solarbio, China) solution. The excess ARS dye was washed with deionized water twice at room temperature. A Zeiss Carr stereo microscope was used to capture the images of calcified nodules on the surface of the PEEK sample. The ARS stained product was incubated in HClO_4_(5 w/v%, Aladdin, Shanghai, China) at 37 °C for 30 min, and calcium nodules could be released and injected into the 5% HClO_4_. And then, the absorbance of the supernatant was measured at 490 nm.

### 2.7. Rna isolation and quantitative real-time PCR (RT-PCR)

In a 24 well plate, MC3T3-E1 cells (3 × 10^4^ cells) were cultured in a plate with the placed PEEK (Φ 10 mm × 2 mm) samples. After the incubation at 37 °C for 3 days and 7 days, the osteogenic-related genes, like ALP, Runx 2 (runt-related transcription factor 2), osteocalcin (OCN), and collagen type 1(COL-I), were measured using RT-PCR, respectively. The standard method for each target gene through the normalization of glyceraldehyde-3-phosphate dehydrogenase (GAPDH) and the calculation using the 2^−ΔΔCt^ method was applied. The primer sequence for each gene is listed in Table S1.

### 2.8. The assay of the immunomodulatory activity

In a 24 well plate, RAW264.7 cells (6 × 10^4^ cells) were cultured on the different PEEK (Φ 10 mm × 2 mm) samples. After 72 h incubation, the concentrations of IL-4, IL-10, TNF-ɑ and IL-6 in the culture medium were measured using respective enzyme-linked immunosorbent assay (ELISA) kit (R & D systems, USA) in accordance to the provided protocol. Moreover, flow cytometry was applied to analysize the molecular markers of CCR7 and CD206 of RAW 264.7 cells after 72 h culture.

### 2.9. *In vivo* experiments

The *in vivo* experiment that approved by the animal ethics committee of Fujian Medical University was carried out based on the manage regulations of experimental animals. Twelve 6-month-old SD rats weighing around 270 g were respectively anesthetized by the mixture of mebendazine (5 mg/kg) and ketamine (65 mg/kg) through intraperitoneal injection. After the hair shaving and surgical towel cover, a hole (d = 2 mm, h = 5 mm) was drilled on the lateral side of the vertical femur with a Kirschner wire (Φ 2 mm). A 30 μL 10^4^ CFU/ml *S. aureus* suspension or saline was injected into femoral cavity. SPEEK, SPEEK-pDA-GS and nature PEEK (Φ 2 mm × 5 mm) were applied as implants in the different operation groups. At the same time, only saline was injected into PEEK (−) and SPEEK (−) groups, and *S. aureus* suspension was injected into the drilling site represented by SPEEK (+) and SPEEK-pDA-GS (+) groups. After operation, rats in each group were fed regularly and separately.

### 2.10. Statistical analysis

With the calculated standard deviation (SD), the statistical analysis was carried out using Graph Pad Prism 7.0. The differences at *p* < .05 (*), *p* < .01 (**), and *p* < .001 (***) were considered as statistical significance.

## 3. Results and discussion

### 3.1. Characterization of different PEEK implants

**Figure 1.**
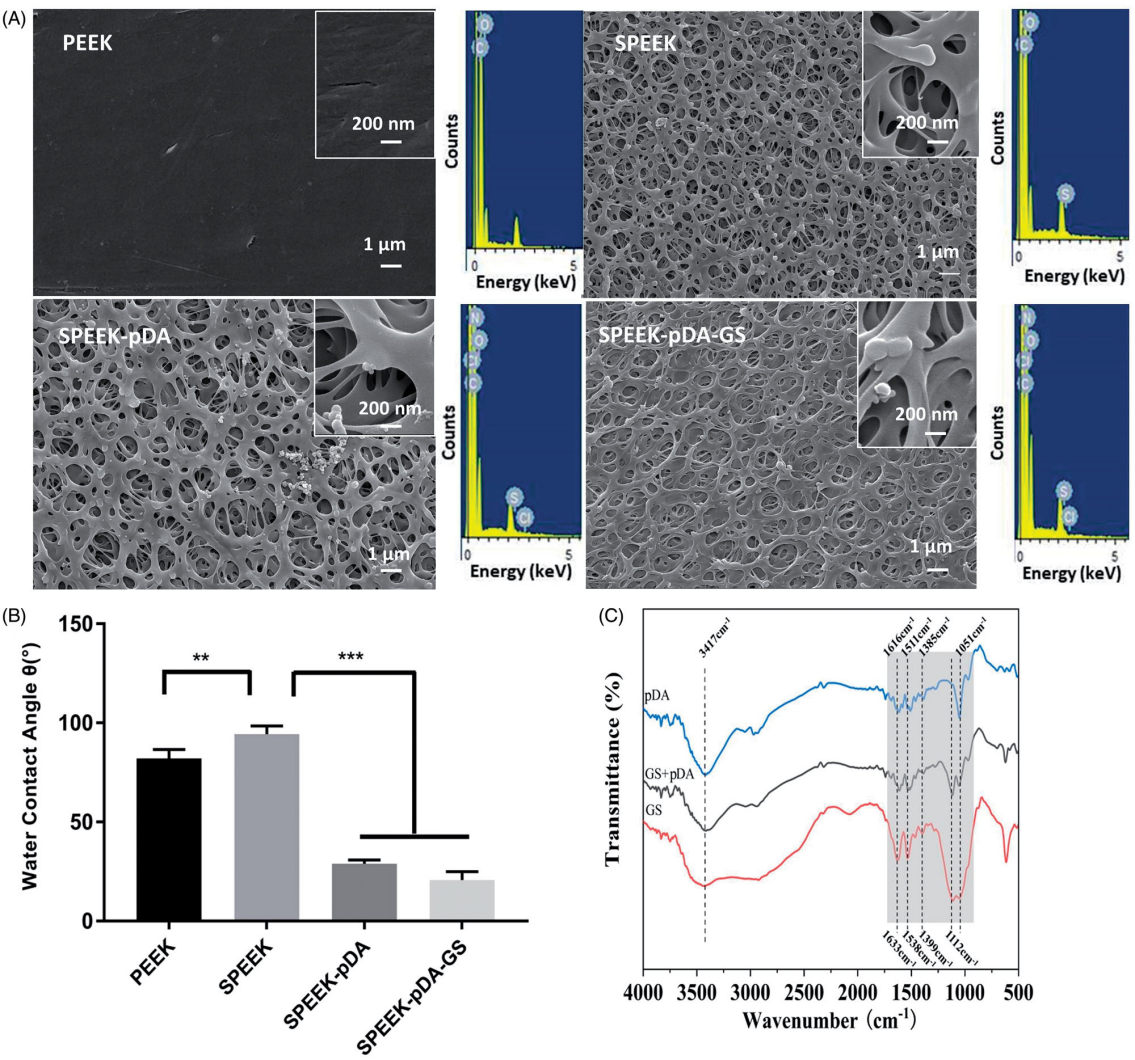
SEM images (A) and water contact angles (B) of PEEK, SPEEK, SPEEK–pDA, and SPEEK–pDA–GS. (C) FTIR spectra of pDA, GS + pDA, and GS. Inset of (A) presents the EDS spectra of the obtained SEM imaging area of the corresponding samples. (*n* = 3, * *p* < .05, ** *p* < .01, and*** *p* < .001).

As shown in Figure 1(A), natural PEEK was relatively smooth with a few slight bumps or lines. And the pore diameter of SPEEK ranged from 23 nm to 728 nm (average pore size was about 173 nm). The axial plane of SPEEK obtained from SEM was applied to investigate the depth of the pores of SPEEK (Figure S1). As illustrated in Figure S1, compared with the smooth morphology of the cross section of PEEK without any pores, the axial plane of SPEEK exhibited the distributed pores with the width from 1 to 7 μm, implied the depth range of the different pores of SPEEK. The SEM results implied that the 3 D structure with pores on SPEEK surface was fabricated. Like SPEEK, the prepared SPEEK–pDA and SPEEK–pDA–GS exhibited the similar porous morphology along with relatively uniform pore diameter, which showed that SPEEK did not destroy its original surface structure during drug loading. Relatively, the pore diameter of SPEEK–pDA–GS ranged from 21 nm to 173 nm, and the average pore size of SPEEK–pDA–GS (55 nm) was smaller than that of SPEEK, suggesting that the successful immobilization of pDA and GS reduce the pore size of SPEEK. In addition, through the EDS analysis, compared with the lack of N content in SPEEK, the existence of N (0.18% elemental content) in SPEEK–pDA, and the increased content of N (0.25% elemental content) in SPEEK–pDA–GS revealed the composition of the prepared activated SPEEK. Furthermore, the water contact angle was evaluated (Figure 1(B)). The surface contact angle of natural PEEK was 82.02°, whereas the surface contact angle of SPEEK increased to 94.27° ascribed to the hydrophobic nature of porous structure. The water contact angle of SPEEK–pDA dropped sharply to 28.89°. Compared with that of SPEEK–pDA, the water contact angle of SPEEK–pDA–GS further dropped slightly to 20.70°, indicating that the surface hydrophilicity of SPEEK–pDA–GS was better than that of SPEEK–pDA and SPEEK. The decreased contact angle of SPEEK–pDA and SPEEK–pDA–GS was attributed to the hydrophilic property of pDA and GS. Moreover, the improved surface hydrophilicity of implant promoted the integration of implant and body bone.

The chemical compositions of pDA and mixture of pDA and GS on SPEEK were further characterized using FTIR. In the pDA FTIR spectrum (Figure 1(C)), the absorption peaks at 3417 and 1616 cm^−1^ were ascribed to –NH_2_ vibration. Moreover, the characteristic absorption peaks at 1511 and 1741 cm^−1^ were attributed to benzene C=C and C=O, respectively, revealing the molecular structure of pDA (Chen et al., 2019). For the GS FTIR spectrum, besides the overlapped wide absorption peak centered at 3459 cm^−1^ ascribed to the –OH and N–H stretching vibration, the peaks at 1633 and 1538 cm^−1^ were ascribed to benzene C=C, and the peak at 1112 cm^−1^ belonged to C–O–C stretching vibration. The pDA–GS spectrum contained the molecular structure characteristics of pDA and GS. These characteristics included the absorption peaks of 3403 cm^−1^ (stretching vibration of –OH), 1614 and 1527 cm^−1^ (stretching vibration of benzene C=C), 1741 cm^−1^ (stretching vibration of C=O), and stretching vibration of C–O–C at 1117 and 1056 cm^−1^. Although no new absorption peak formed in the co-deposition of pDA and GS, the slight shift in the absorption peaks of C–O–C and C=C exhibited the interaction between pDA and GS in the modification process of SPEEK–pDA–GS (Pei, 2015). Furthermore, compared with the absorption peak of –OH in pure GS, the red shift of –OH ascribed to GS in SPEEK–pDA–GS suggested that one of the interaction between pDA and GS was intermolecular hydrogen bonding (Pei, 2015).

The results of the compression test are shown in the Fig. S2. The typical load–displacement curves of different PEEK samples were observed. PEEK, SPEEK, SPEEK-pDA and SPEEK–pDA–GS could withstand the maximum load of 8000 Newtons, the maximum compressive strength that the machine could measure. Moreover, the three treated PEEKs exhibited smaller deformation distance than the PEEK under the similar load strength. This result suggested that the material of the porous surface and the modification of its surface did not change the PEEK’s original compressive mechanical strength without any deformation, suggesting the good mechanical strength of the modified PEEK sample.

**Figure 2.**
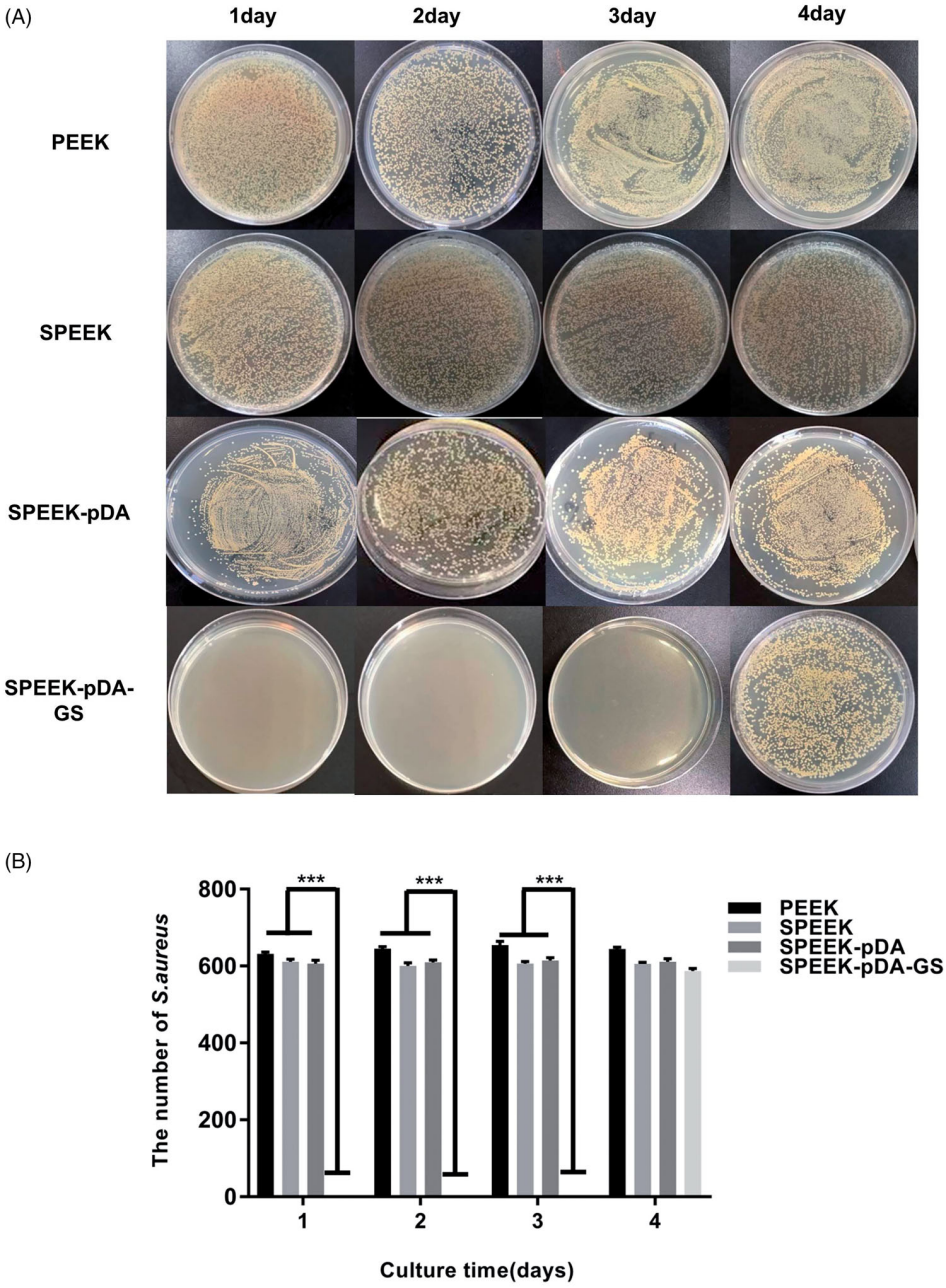
Images (A) and the number of the bacterial colonies (B) of *S. aureus* added, isolated, and cultured on agar for consecutive days with the co-culture of natural PEEK, SPEEK, and SPEEK–pDA–GS. (*n* = 3, *** *p* < .001).

The release curve of GS from SPEEK–pDA–GS was tested in SBF at 37 °C and different times (Figure S3). The SPEEK–pDA–GS release process was relatively fast. In the open environment, the total release of GS was less than 72 h, and the concentration of GS peaked at SPEEK–pDA–GS was nearly 12 h. The cumulative release of the all loaded GS was about 10.13 μg. The sustained GS release of the SPEEK–pDA–GS implied the antibacterial ability of SPEEK–pDA–GS. Moreover, this result suggested that the simple one-step deposition of pDA could simultaneously load moderate mass of GS onto the porous SPEEK.

**Figure 3.**
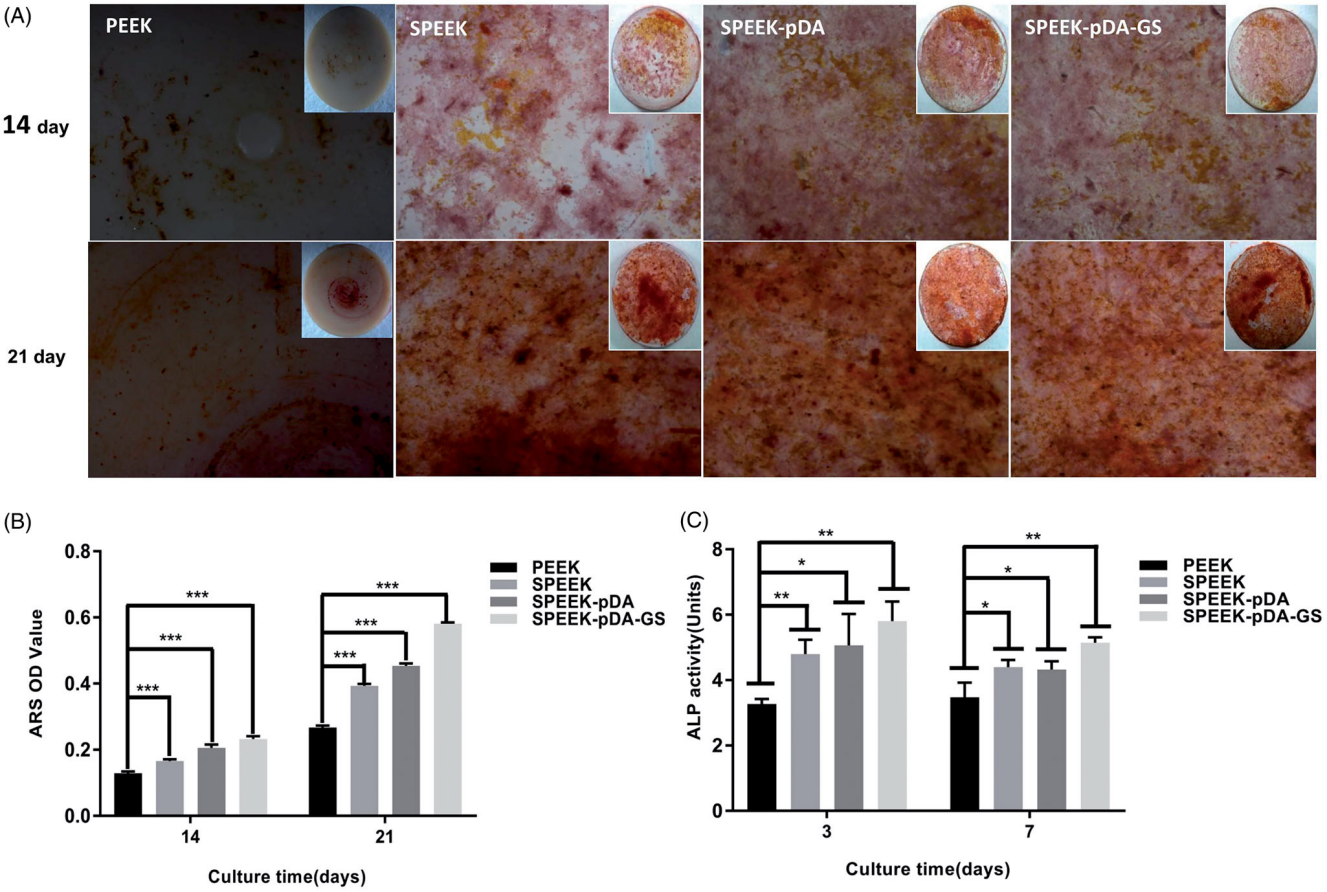
Effects of PEEK, SPEEK, SPEEK–pDA, and SPEEK–pDA–GS on the osteogenic differentiation of MC3T3-E1 under osteoinductive and osteoconductive conditions. (A) ARS staining for the morphology evaluation of the calcium deposition and (B) calcium deposition results on days 14 and 21. (C) Determination of ALP activity on days 3 and 7. (*n* = 3, * *p* < .05, ** *p* < .01, and *** *p* < .001).

### 3.2. *In vitro* antibacterial ability

The potential persistent antibacterial properties of different PEEK samples were compared in a consecutive period. At the first 24 h, numerous colonies suggested that PEEK, SPEEK and SPEEK-pDA plates exhibited almost no bactericidal ability on *S. aureus* or *E. coli*, as shown in Figure S4(A). Accordingly, SPEEK–pDA–GS showed strong antibacterial activity on day 1, reaching almost 100% against *S. aureus* and *E. coli* without any colony (Figure S4(B)). Figure S5 illustrates live/dead staining results of *S. aureus* and *E. coli* on the three selected samples. Much more live bacteria appearing as green fluorescence was found on bare PEEK and SPEEK than that on SPEEK-pDA-GS, suggesting that almost no live bacteria absorbed on SPEEK-pDA-GS. These findings confirmed that the prepared SPEEK–pDA–GS possessed the antibacterial activity ascribed to the co-deposition of GS with the formation of pDA.

**Figure 4.**
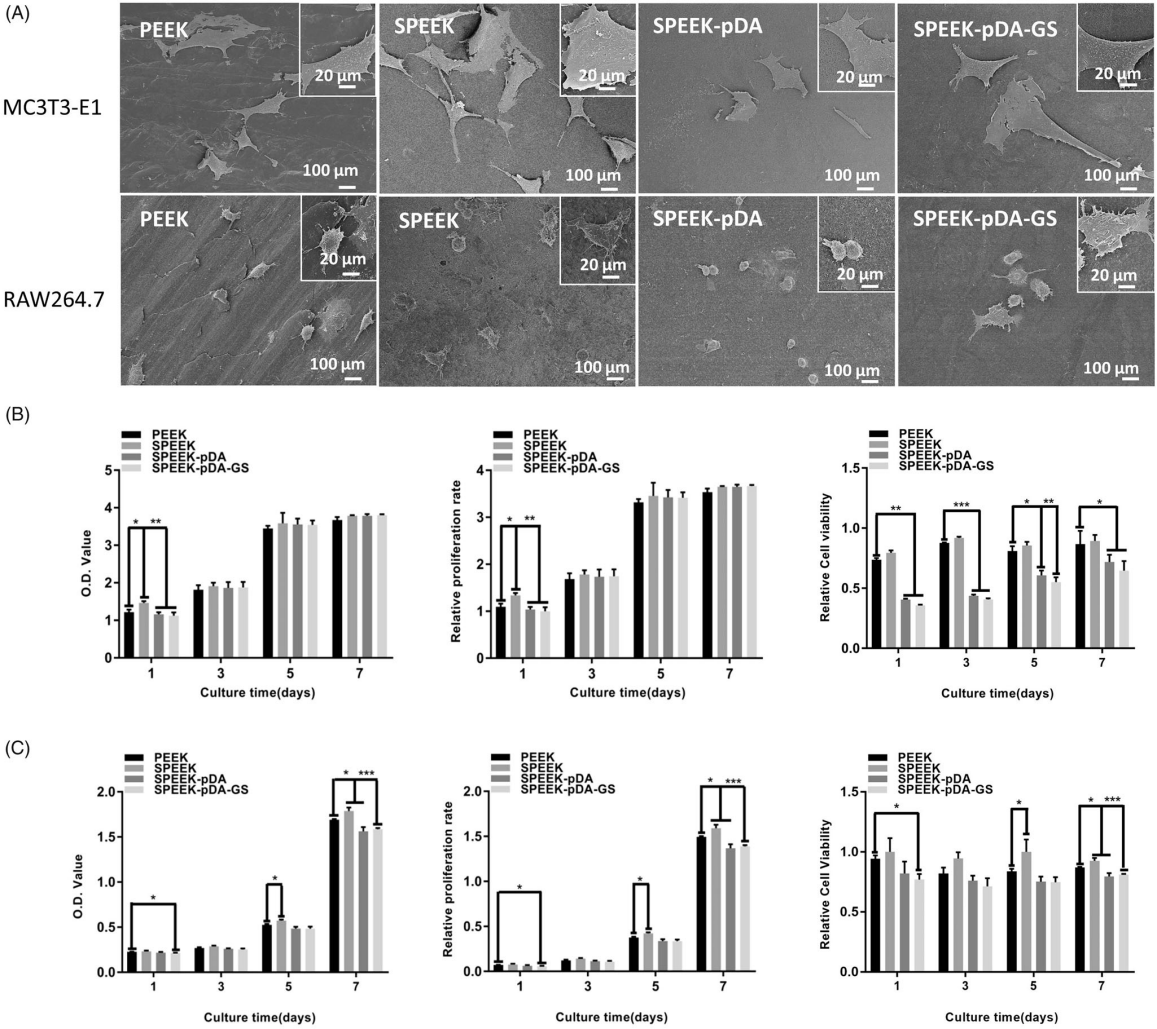
(A) SEM images of MC3T3-E1 and RAW264.7 cells after being cultured on different samples for 24 h at low and high magnifications. OD value, proliferation ability, and cell viability of MC3T3-E1 (B) and RAW264.7 (C) cells in different groups on days 1–7 detected using the CCK-8 assay. The value of 1 in the Y-axis of the third column of (B) and (C) is the cell viability in plate without any sample interference. (*n* = 3, * *p* < .05, ** *p* < .01, and *** *p* < .001).

**Figure 5.**
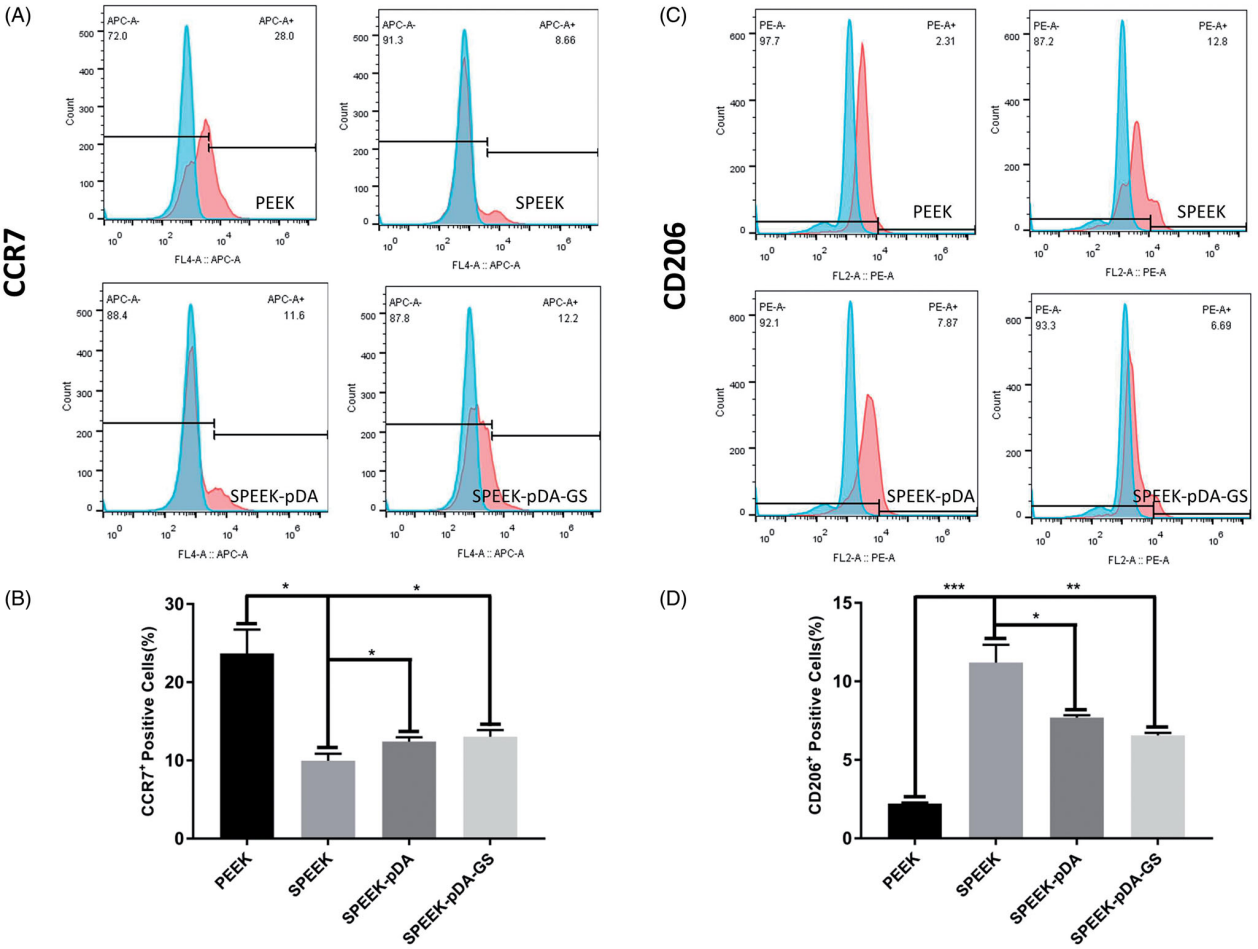
(A, C) Representative images of surface markers (CCR7 and CD206) of RAW264.7 analyzed using flow cytometry, and (B, D) the percentages of CCR7- or CD206-positive cells on PEEK, SPEEK, SPEEK–pDA, and SPEEK–pDA–GS, respectively. (*n* = 3, * *p* < .05, ** *p* < .01, and *** *p* < .001).

In addition, the continuous antibacterial abilities of different modified PEEKs were evaluated. PEEK, SPEEK and SPEEK-pDA had no antibacterial activity throughout the process. Almost no colony was observed in SPEEK–pDA–GS for the initial 3 days (Figure 2(A)), indicating that SPEEK–pDA–GS had effective and extended antibacterial properties. SPEEK–pDA–GS lost its antibacterial activity on day 4 ascribed to the little content of residual GS on SPEEK–pDA–GS. Although SPEEK–pDA–GS rapidly released GS in the SBF solution (Figure S3), *in vitro* co-culture results showed that SPEEK–pDA–GS effectively inhibited bacteria for three consecutive days. The persistent bacterial clearance implied the potential of SPEEK–pDA–GS as an implant to combat possible bacterial infection.

### 3.3. Osteogenic ability of different PEEK samples

The osteogenic abilities of modified PEEK samples were evaluated. At first, the calcium deposition on the modified PEEK samples were carried out and compared to evaluate the osteogenic efficiency using ARS staining. As shown in Figure 3(A), calcium nodules were formed on all PEEK samples on days 14 and 21. Moreover, MC3T3-E1 cells cultured on the SPEEK–pDA–GS surface on day 14 were able to form distinct small bone nodules. On day 21, increased and compact bone nodules were formed on the different samples. For SPEEK–pDA–GS, densely distributed nodules were discovered on day 21. By comparison, gradually increased calcium nodules were formed on PEEK, SPEEK, SPEEK–pDA, and SPEEK–pDA–GS, suggesting the enhanced osteogenic ability of modified PEEK. The same trend was observed on day 14. These results were in accordance with the quantitative calcium deposition (Figure 3(B)). As shown in the quantitative analysis, the SPEEK–pDA–GS sample had more nodules than the rest of the group. The ARS staining illustrated the acceptable induced bone differentiation of SPEEK–pDA–GS for a long time. In SPEEK–pDA–GS, pDA and GS loading on the SPEEK surface increased the hydrophilicity of the sample surface, stimulated the osteogenic potential, and improved the osteogenic efficiency, which was reflected by the dense mineralized nodules. Therefore, the combination of pDA and GS, which were two hydrophilic drugs, might play a positive synergistic effect in promoting bone formation in the cell culture medium.

Furthermore, the cell differentiation of MC3T3-E1 cells on different samples was evaluated through the measurement of alkaline phosphatase (ALP) activity (Figure 3(C)). Compared with that of the natural PEEK group, the ALP activity of the SPEEK–pDA–GS group was increased on day 3 (*p* < .01). On day 7, although the ALP activity was relatively reduced in all groups, the ALP activity in SPEEK–pDA–GS group was still the highest in all groups. The ALP result implied the ability of SPEEK–pDA–GS to promote bone formation, which was in accordance with that of calcium deposition.

Furthermore, the expression of osteogenic genes in MC3T3-E1, like ALP, Runx2, OCN, and Col-I, was determined using RT-PCR after co-culture with different samples on days 3 and 7 (Figure S6). After three days in culture, the upregulation of four osteogenic genes in MC3T3-E1 followed the order: PEEK < SPEEK < SPEEK–pDA < SPEEK–pDA–GS. The same trend was still upward on day 7, suggesting that modified PEEK promoted the cell differentiation. Consistency was observed between the results of the ARS staining and the ALP quantitative analysis.

**Figure 6.**
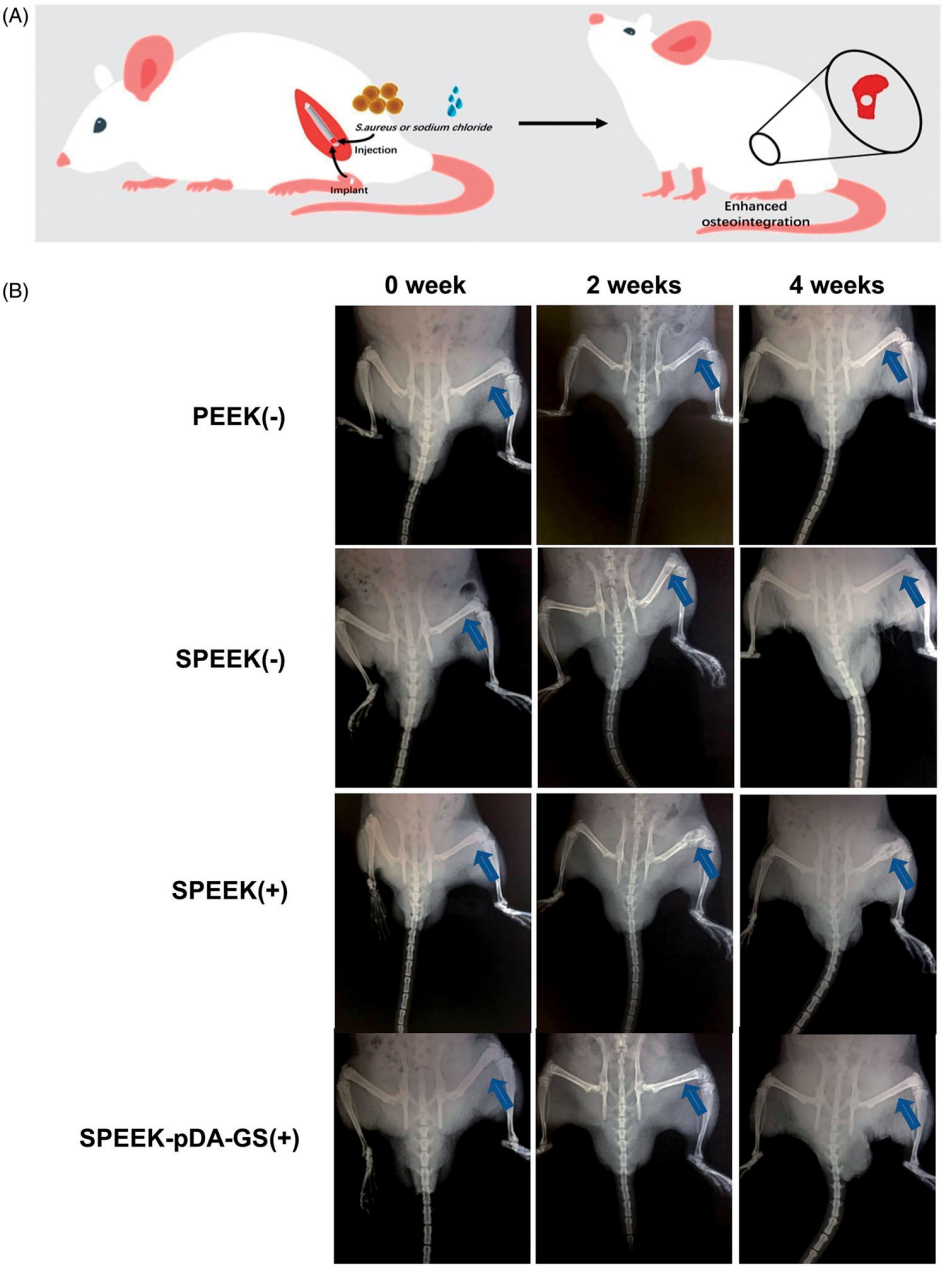
(A) Schematic illustration of the *in vivo* process of the implant for the uninfected (-) or infected (+) rat. (B) X-ray images of rats of PEEK (-), SPEEK (-), SPEEK (-), and SPEEK–pDA–GS (+) groups at 0, 2, and 4 weeks after surgery. The blue arrow indicates the implantation site.

### 3.4. Cell viability of MC3T3-E1 and RAW264.7 cells on different PEEK samples

The cell viability on different PEEK samples was further studied to evaluate the *in vitro* biocompatibility and osteogenic differentiation of SPEEK–pDA–GS. SEM images (Figure 4(A)) showed that the cells adhered well on different groups of samples. The cells were fusiform on the natural PEEK and did not expand completely. When cultured on SPEEK, SPEEK–pDA, and SPEEK–pDA–GS, the morphologies of MC3T3-E1 and RAW264.7 expanded completely. In addition, many cellular pseudopodia were found on the surfaces of the three modified PEEK samples. Furthermore, the both MC3T3-E1 and RAW264.7 of the SPEEK, SPEEK–pDA, and SPEEK–pDA–GS groups were more closely interconnected and cross-sectional than those cells of the natural PEEK group. This result suggested that the modification of pDA and GS did not inhibit the growth but promoted the flat diverse distribution of cells on modified PEEK samples. Furthermore, the SEM image of cells inoculated for 24 h on different surfaces indicated that the adhesion of bone cells and macrophages to the modified PEEK was better than that to the pure PEEK. In addition, compared with those on the natural PEEK, the RAW 264.7 cells on the modified PEEK samples seemed to be spread more evenly and more diversely (Figure 4(A)), suggesting the specific inducement effect of porous surface structure (Liu et al., 2019; Wei et al., 2019).

In addition, the CCK-8 kit was further used to assess the cell proliferation on different modified PEEKs (Figure 4(B,C)). Given that the proliferation ability of RAW264.7 cells was stronger than that of MC3T3-E1, the initial cell seeding number of RAW264.7 was only half of the number of MC3T3-E1 cells to meet the need of the test well. In the 7-day trial period, MC3T3-E1 cells exhibited gradually increasing proliferative behavior in all samples. Moreover, the proliferation of cells on SPEEK–pDA–GS increased gradually in the 7-day experiment. Although the potential negative effect of relatively high concentration of GS on cell (Chang et al., 2006), the cell proliferation ability on SPEEK–pDA–GS with the prepared loading content of GS was comparable with that on other groups. The MC3T3-E1 cell activity decreased at the initial 1 days but increased evidently after three days on SPEEK–pDA–GS, which exhibited comparable viability with other groups without GS. This result may be due to the negative effect on cells from the initially high released concentration of GS. Correspondingly, the growth pattern of RAW264.7 cells on different samples was basically the similar as that of MC3T3-E1 cells with a certain disturbing. It is necessary to point out that the small number of RAW264.7 cells may induce RAW264.7 did not proliferate significantly until day 5. On day 7, cells showed a leaping growth. Combined with the SEM results, cell proliferation tested from CCK-8 implied that even with the introduction and potential release of GS, SPEEK–pDA–GS still exhibited acceptable biocompatibility for the promotion of cell growth of MC3T3-E1 and RAW264.7 cells. These results might be due to the porous structure and hydrophilicity of SPEEK–pDA–GS with appropriate roughness, which might be advantageous for subsequent cell adhesion and proliferation (Sola-Ruiz et al., 2017; Fu et al., 2021) to reduce the negative impact of released GS in the cell growth process for *in vivo* application.

### 3.5. Macrophage polarization of different PEEK samples

Besides the antibacterial ability and osteogenic ability, the potential immunoregulatory behavior of modified PEEK was investigated and compared. The representative cytokines secreted by anti-inflammatory M2 and proinflammatory M1 phenotype macrophages were tested using ELISA (Figure S7). The macrophages on SPEEK secreted the highest amount of anti-inflammatory cytokines IL-4 and IL-10, which were produced by M2 type macrophages (Liu et al., 2018). Although the IL-4 and IL-10 on SPEEK–pDA and SPEEK–pDA–GS were somewhat lower than those on SPEEK, these anti-inflammatory cytokines were still significantly higher than that secreted by macrophages on pure PEEK (*p* < .01). By contrast, two inflammatory cytokines of TNF-α and IL-6 produced by M1 macrophages on SPEEK were expressed lower than that on PEEK. Moreover, the further prepared SPEEK–pDA and SPEEK–pDA–GS exhibited further reduction of TNF-α and IL-6, suggesting that the proinflammatory ability secreted by M1 macrophages was significantly weakened on SPEEK–pDA and SPEEK–pDA–GS.

**Figure 7.**
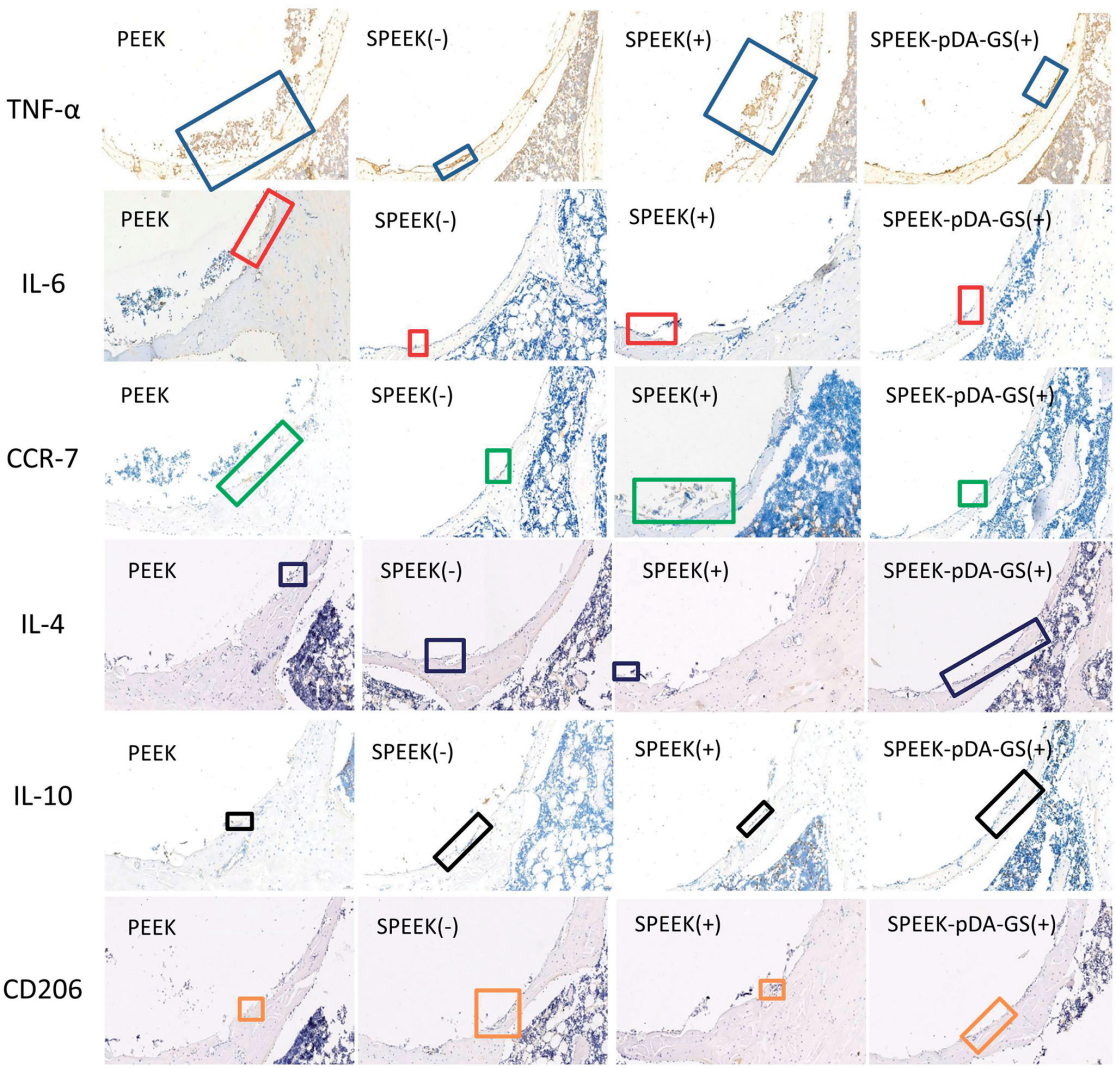
IHC staining for anti-inflammatory (IL-4 and IL-10) and proinflammatory (IL-6 and TNF-α) proteins and surface markers (CCR7 and CD206) of macrophage cells from the in vivo experiments of the PEEK (-), SPEEK (-), SPEEK (+), and SPEEK–pDA–GS (+) groups.

Furthermore, the flow cytometry was applied to determine the percentage of M1 or M2 macrophage cells on modified PEEK samples through the simultaneous analysis of the molecular markers of CCR7 and CD206 for M1 and M2 type macrophages (Liu et al., 2018), respectively (Figure 5). The percentage of CCR7-positive cells dropped from 23.7% in PEEK group to 9.95% in SPEEK group, 12.4% in SPEEK–pDA, and 13.03% in SPEEK–pDA–GS. In addition, compared with the percentage of the expression of CD206 on SPEEK group (11.2%), the percentages of the M2 phenotype marker CD206 in the SPEEK–pDA (7.69%) and SPEEK–pDA–GS (6.56%) groups were higher than that in the PEEK group (2.22%), indicating the immunoregulatory ability of functional PEEK of the controllable induction of macrophages to M2 phenotype. The SPEEK–pDA–GS had a certain degree of immunoregulatory ability of inducing macrophages to the M2 phenotype. The immunoregulatory ability of SPEEK–pDA–GS may be due to its characteristics of porous structure and hydrophilicy (Liu et al., 2019). Therefore, SPEEK–pDA–GS with porous structure and appropriate GS loading mass could regulate and achieve the appropriate balance among antibacterial activity, cell biocompatibility, and controlled immunoregulatory of anti-inflammatory M2. For the potentially coexisting bacterial reproduction, inflammation, and immunosuppression in infected bone destruction (Ram and Chinen, 2011; Liu et al., 2019), such balanced properties of antibacterial ability and immune regulation of SPEEK–pDA–GS will effectively treat bacterial infection and alleviate the inflammatory response to enhance the needed osseointegration for *in vivo* implanting to infected bone destruction.

### 3.6 X-Ray and blood routine examination

The role of the prepared SPEEK–pDA–GS in promoting osseointegration was explored by establishing an *in vivo* model of bone destruction with infection (Figure 6(A)). For comparison, infected group treated with SPEEK without drug loading and uninfected groups treated with implant were applied. X-rays were used to evaluate the potential disease progression at the surgical site within a specified time. Figure 6(B) shows the dynamic changes in the surgical site at 0, 2, and 4 weeks after implanting. However, PEEK is a radiolucent implant material, which means that it does not produce artifacts in tomograms and magnetic resonance images. The implant shadowed as a circular hole could be found at the position indicated by the blue arrow (Figure 6(B)). Imaging signs of local bone destruction caused by surgery could be found at the initial stage of surgery. In the experimental process, no low-density shadow around the relatively regular implant site was achieved in the uninfected groups (PEEK (−) and SPEEK (−) groups), indicating that the PEEK implant could promote osseointegration without infection. In the infection group treated by SPEEK (SPEEK (+) group), patchy bone destruction areas could be seen in the cancellous bone around the implant, and the trabecular bone structure was blurred. The edges of the destruction area were also blurred. The accumulated cortical bone invaded most of the bones and was complicated by disease. Four weeks after the operation, the implant area (SPEEK (+) group) exhibited the characteristics of periosteal hyperplasia, thickening of the cortex, widening of the medullary cavity, thickening of the diaphysis, further destruction of bone accompanied by osteoporosis, the existence of dead bones and dead space, and even chronic osteomyelitis. These imaging changes suggested the uncontrollable infection and bone destruction with the SPEEK (+) group. In the infection group treated by SPEEK–pDA–GS (SPEEK–pDA–GS (+) group), no imaging change similar to acute purulent osteomyelitis was found around the implantation site after two weeks and then completely healed without any space or abscess at the fourth week after operation. Then, the femur at the surgical site was separated and compared (Figure S8). In groups without infection (i.e., PEEK (−) and SPEEK (−) groups), dry field of vision in the surgical area, no abscess oozing, and tight connection of the implant to the peripheral bone tissue were found. In the SPEEK (+) group, pus around the implantation area, thickened bone shaft, irregular shape, large gap that could easily loosen between the implant and the surrounding bone were observed. By contrast, the SPEEK–pDA–GS (+) group exhibited that the infection did not spread on the bone surface with the good integration between implant and bone. Furthermore, the white blood cell count (Figure S9) was measured to evaluate the body reaction to assess the recovery of this *in vivo* process. The white blood cells in the SPEEK (+) group increased obviously compared with those in two other groups without infection or the SPEEK–pDA–GS (+) group in the process from day 7, suggesting that the local infection might spread and induce systemic infection. For the SPEEK–pDA–GS (+) group, the white blood cell counts had no significant difference with those in the two groups without infection. Combined with the X-ray images and white blood cell analysis, the early acute suppurative osteomyelitis in the SPEEK (+) group was not well treated, and the infection could easily turn into chronic osteomyelitis and even cause systemic inflammation. In the SPEEK–pDA–GS (+) group, the infection was locally controlled and then cured, which was ascribed to the enough and sustained release of GS in a certain period (Javier et al., 2019).

**Figure 8.**
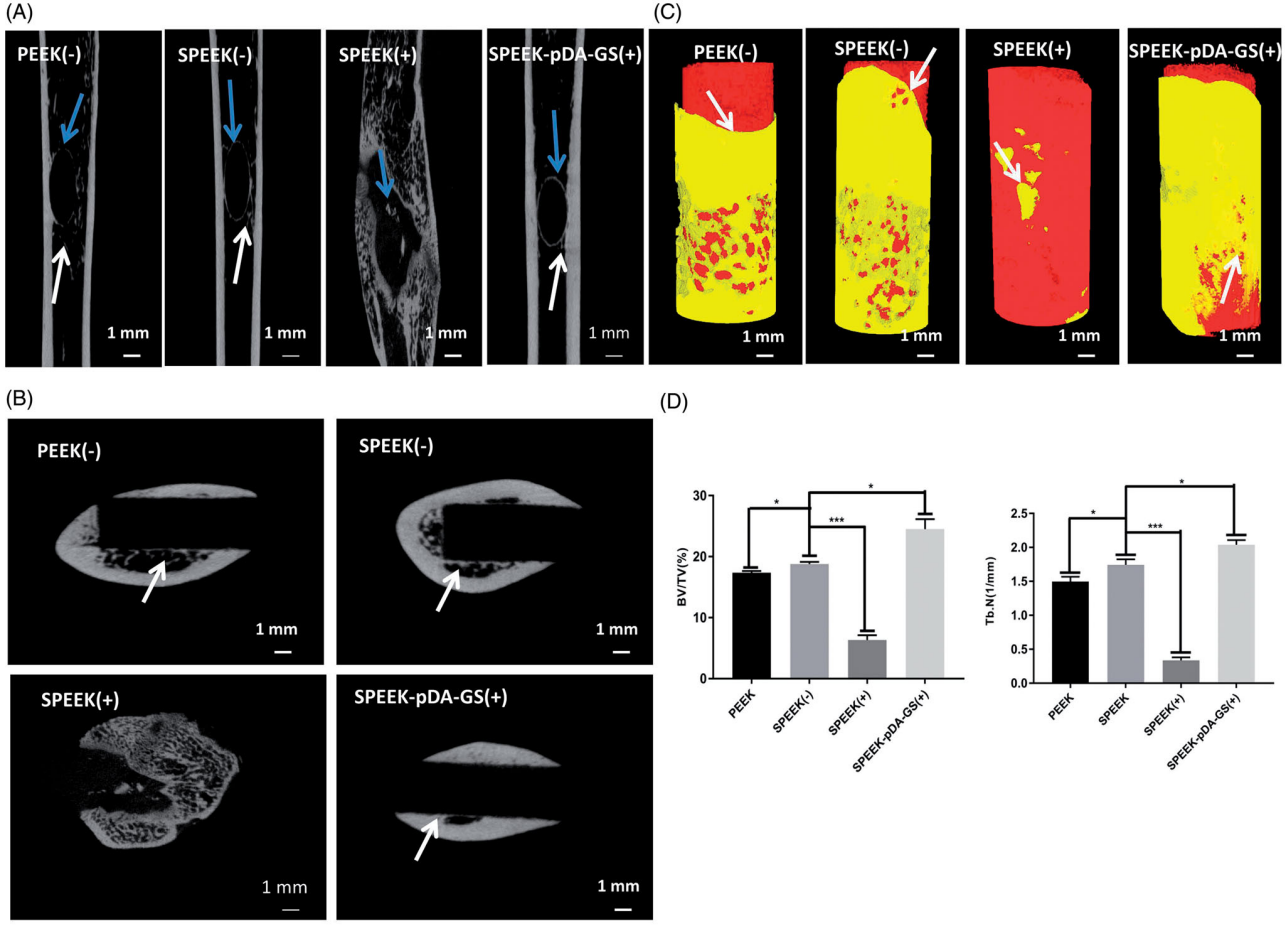
(A) Horizontal axis image of the implant. Blue arrows show the new bone around the implant, and white arrows show the trabeculae. (B) Vertical axis image of the implant. White arrows show where the new bone is attached to the implant. (C) 3 D reconstruction images of different samples of bone implants. The white arrow indicates the interface where the implant connects to the surrounding bone. (D) Quantitative analysis of (a) BV/TV and (b) Tb.N after implantation for six weeks with different modified PEEK samples. The implantation rats were PEEK (-), SPEEK (-), SPEEK (+), and SPEEK–pDA–GS (+) groups, respectively. (*n* = 3, * *p* < .05, ** *p* < .01, and *** *p* < .001).

**Figure 9.**
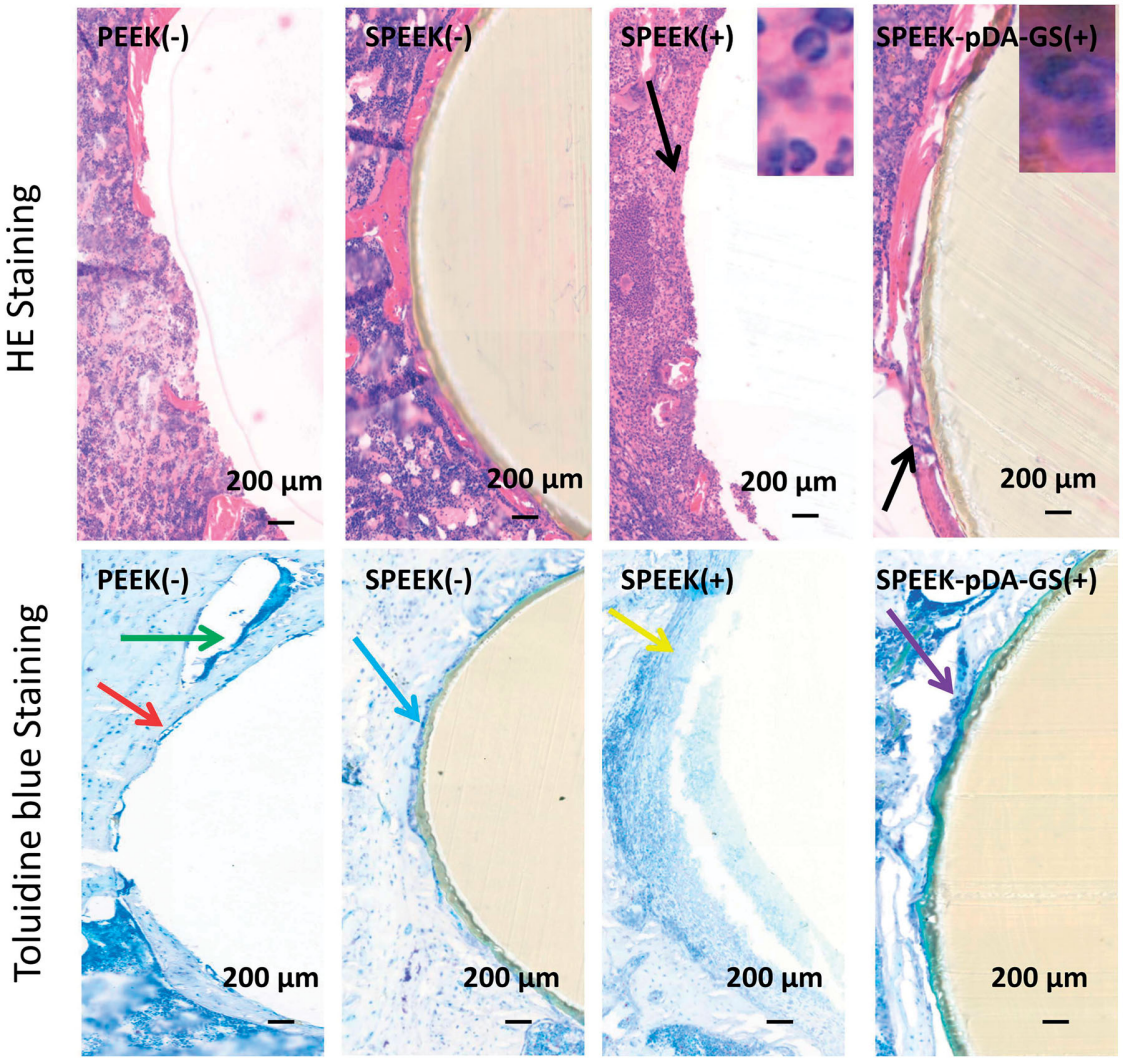
Histological analysis of the *in vivo* bone integration using HE and toluidine blue staining methods in PEEK (-), SPEEK (-), SPEEK (+), and SPEEK–pDA–GS (+) groups, respectively.

At six weeks after operation, the femoral heads of rats in different groups were collected for immunohistochemical analysis (Figure 7). The expression of the proinflammatory factor TNF-α (blue rectangle) in the PEEK (−) and the SPEEK (+) groups was stronger than that in the SPEEK (−) and the SPEEK–pDA–GS (+) groups. Similarly, the expression levels of proinflammatory factors IL-6 (red rectangle) and CCR7 (green rectangle) in the PEEK (−) and SPEEK (+) groups were stronger than those in the SPEEK (−) and SPEEK–pDA–GS (+) groups. The anti-inflammatory factors IL-4 (blue rectangle), IL-10 (black rectangle), and CD206 (orange rectangle) in SPEEK (−) and SPEEK–pDA–GS (+) groups were weak but were still stronger than those in the PEEK (−) and SPEEK (+) groups. Therefore, compared with the expression of the PEEK (−) group, the SPEEK (−) and SPEEK–pDA–GS (+) groups could enhance the expression of anti-inflammatory factors and weaken the expression of proinflammatory factors in *in vivo* experiments. In addition, the SPEEK–pDA–GS(+) group could effectively control local infection and had improved anti-inflammatory expression in the implant site. This result was consistent with the *in vitro* results, which showed that SPEEK–pDA–GS had antibacterial and immunomodulatory effects.

### 3.7. Bone formation using μ-CT evaluation

The μ-CT was used to investigate the local and the peripheral bone tissues of femur after implantation and reconstruction (Figure 8). The integration of implant and bone tissue was directly evaluated from the horizontal axis of the implant (Figure 8(A)). In the PEEK (−) group, the discontinuous new bone was formed around the implant with some defects. In the SPEEK (+) group, only a small amount of new bone was formed with discontinuous shape. Compared with the PEEK (−) and the SPEEK (+) groups, the implants in the SPEEK (−) and SPEEK–pDA–GS (+) groups had complete new bone (blue arrows) around implant, which also formed a “bridge” for the relation of the implant and the bone, known as the trabecular bone (white arrow). The new formed bone trabeculae indicated that the implant could be combined with the surrounding bone tissue. Figure 8(B) represents the lateral bone integration characteristics of the implant and the peripheral bone. The white arrow represented the interface between the implant and the surrounding bones. The bone tissue around the implants in the SPEEK (+) group was atrophic, and some evident gaps were formed, which indicated that the bone integration was poor. Few and discontinuous new bones were found around the implant. Although the formed new bone and a certain degree of integration around the implant was found, the bone mass and the osteointegration degree in the PEEK (−) group were somewhat lower than those in the SPEEK (−) and SPEEK–pDA–GS (+) groups. The excellent bone integration with continuous new bone was found around the implant of the SPEEK (−) group or the SPEEK–pDA–GS (+) group. Figure 8(C) shows the local femur of the implant reconstructed with μ-CT. The white arrow indicated the new formed interface for the implant. The bone tissue around the natural PEEK formed some tiny gaps in PEEK (−) group, whereas the surrounding bone tissue in the SPEEK (−) group grew, adhered, and wrapped along the surface of the implant, showing the characteristics of strong osseointegration. Although some new bone forms were found in the SPEEK (+) group, the new bone mass and the new formed interface around implant were significantly reduced. The implant did not connect with the surrounding bone tissue, suggesting that the ineffective control of local infection induced the formation of necrotic bone. By contrast, in the SPEEK–pDA–GS (+) group, no bone destruction was observed, but the freshly formed bone tissue tightly attached to the bone tissue. This finding indicated that the SPEEK–pDA–GS could cure the local infection well, promote the biological activity of the modified PEEK, and significantly enhance the bone integration. Finally, the bone volume/total volume (BV/TV) and trabecular number (Tb.N) were analyzed quantitatively using 3 D reconstruction results (Figure 8(D)). Compared with those of the PEEK (−) group, the BV/TV and Tb.N of the SPEEK (−) group were significantly higher than those of the PEEK (−) group, suggesting that increased surface roughness improved the BV/TV in the implant group without infection. However, the BV/TV and Tb.N of the SPEEK (+) group were significantly lower than those of all groups, revealing that the presence of infection without control would hinder the osseointegration of implant. The BV/TV and Tb.N of the SPEEK–pDA–GS (+) group was enhanced compared with those of other groups. This result confirmed the considerable osseointegration promotion ability of SPEEK–pDA–GS through the proof of the evident new bone and trabecular bone formation even in the implant area with infection.

### 3.8. Histopathological evaluation

The histological section subjected to HE and toluidine blue staining was carried out at six weeks after implantation (Figure 9). In the HE staining, many scattered neutrophils (black arrow) were seen around the implant in the SPEEK (+) group, indicating that the infection around the implant was not controlled. In the toluidine blue staining, no evident new bone formation but a discontinuous loose bone tissue (yellow arrow) was observed in the SPEEK (+) group. In addition, the implant was hard to keep during the sample preparation process for histopathological evaluation, suggesting that the implant could not be integrated with the bone due to loosening. This finding indicated that if an infection was present, the osseointegration was affected, and the implant could loosen or fall off. The staining of the PEEK (−) and SPEEK (−) groups showed no evident distribution of neutrophil aggregation around the implant. The toluidine blue staining indicated the formation of a certain amount of discontinuous new bone around the implant in the PEEK (−) group (red arrow), which should be improved to integrate the implant with the surrounding bone tissue. The toluidine blue staining showed some new and continuous bone tissues surrounding the implant in the SPEEK (−) group (blue arrow) and that some fibrocysts were formed around the implant due to immune response (green arrow) in the PEEK (-) group. In the SPEEK–pDA–GS(+) group, the HE staining showed that a little amount of neutrophil scattered around the implant, indicating the controlled state of the local bone infection. Moreover, the toluidine blue staining showed that a large amount of continuous new bone tissue was formed around the implant (purple arrow), and the implant was immediately connected to the surrounding bone, suggesting enhanced osseointegration ability.

The histological analysis was performed on the liver and kidney in each group to investigate the implants’ systemic biosafety (Figure S10). No abnormal phenomenon and immune reaction was observed in the corresponding organs under the microscope. The SPEEK loaded with or without pDA and GS showed no harm to the main two metabolic organs of rats, showing good *in vivo* biological safety.

## 4. Conclusions

In summary, the porous PEEK (SPEEK) with a certain diameter and depth was prepared and applied to further modification of GS to introduce biologically inert PEEK the enhanced ability for osseointegration. SPEEK loaded with pDA and GS (SPEEK–pDA–GS) was prepared on the prepared SPEEK surface by a simple one-step co-deposition method. SPEEK–pDA–GS showed biocompatibility with sustained antibacterial activity *in vitro* and promoted the secretion of osteogenic marker and immune regulation function to the macrophage M2 phenotype. Combined with *in vivo* experiments, the SPEEK–pDA–GS could effectively control infection and promote the development of inflammation in favor of tissue repair. The SPEEK–pDA–GS could promote the formation of new bone *in vivo*, and this phenomenon was conducive to the fusion of new bone tissue and implant. Results showed that the SPEEK–pDA–GS had significant antibacterial, anti-inflammatory, and osseointegration effects for the treatment of infectious bone defect. This method has great potential in the rapid and simple surface modification of dental implants and orthopedics.

## Supplementary Material

Supplemental Material
