# Supplementary material for: Facile surface functional polyetheretherketone with antibacterial and immunoregulatory activities for enhanced regeneration toward bacterium-infected bone destruction
Source: Drug Deliv. 2021 Aug 2;28(1):1649–63. doi: 10.1080/10717544.2021.1960924 (PMC8330770; doi:10.1080/10717544.2021.1960924)
Supplement: Supplemental Material [file IDRD_A_1960924_SM7028.docx]

Supplementary Materials

**Facile surface functional polyetheretherketone with antibacterial and immunoregulatory activities for enhanced regeneration towards bacterium-infected bone destruction**

An’an Sun^a,#^, Xi Lin^b,#^, Zhiqiang Xue^a^, Jiyue Huang^a^, Xinxin Bai^a^, Lingling Huang^d^, Xinhua Lin^c^, Shaohuang Weng^c,*^, Min Chen^a,*^

a Department of Orthopedic Surgery, Fujian Medical University Union Hospital, Fuzhou 350001, China

b Department of Emergency Surgery, Center for Trauma Medicine, The First Affiliated Hospital of Fujian Medical University, Fuzhou 350005, China

c Department of Pharmaceutical Analysis, School of Pharmacy, Higher Educational Key Laboratory for Nano Biomedical Technology of Fujian Province, Fujian Medical University, Fuzhou 350122, China

d Department of Stomatology, The First Affiliated Hospital of Fujian Medical University, Fuzhou 350005, China

# Both of these authors contributed equally to this work.

Correspondence: [shweng@fjmu.edu.cn](mailto:shweng@fjmu.edu.cn) (S. Weng); [chenminfz006@163.com](mailto:chenminfz006@163.com) (M. Chen)

Table S1. The primers used in this work for the quantitative real-time reverse transcriptase polymerase chain reaction (RT-qPCR).

| Gene target | Sequence |
| --- | --- |
| GAPDH | Forward:5'-AAGAGGGATGCTGCCCTTAC-3' |
|  | Reverse:5'-CGGGACGAGGAAACACTCTC-3' |
| ALP | Forward: 5'-CTGGACTTGGTGGTCACAGC-3' |
|  | Reverse:5'-AATTGACGTTCCGATCCTGAGT-3' |
| RUNX2 | Forward: 5'-CGCCTCACAAACAACCACAG-3' |
|  | Reverse:5'-TGCTTGCAGCCTTAAATATTCCTG-3' |
| Colla1 | Forward: 5'-ACGCCATCAAGGTCTACTGC-3' |
|  | Reverse:5'-ACTCGAACGGGAATCCATCG-3' |
| OCN | Forward: 5'-CTGACCTCACAGATCCCAAGC-3' |
|  | Reverse:5'-TGGTCTGATAGCTCGTCACAAG-3' |


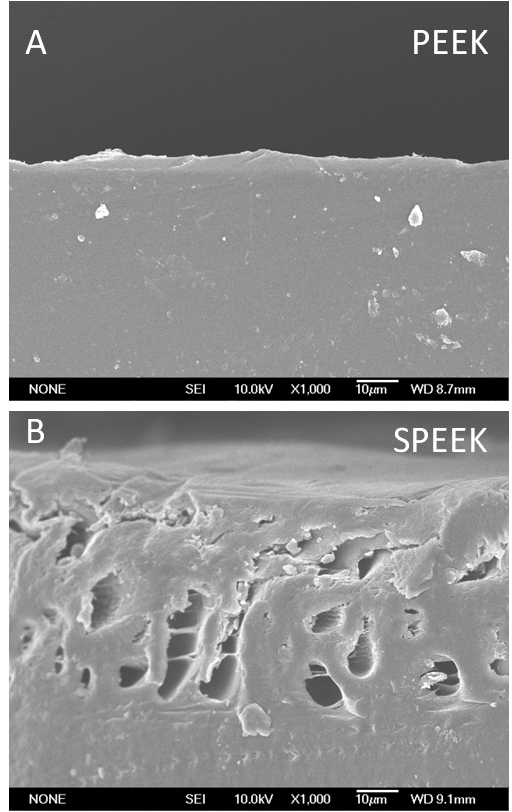


Fig. S1. The SEM images of the cross section of the PEEK (A) and SPEEK (B).


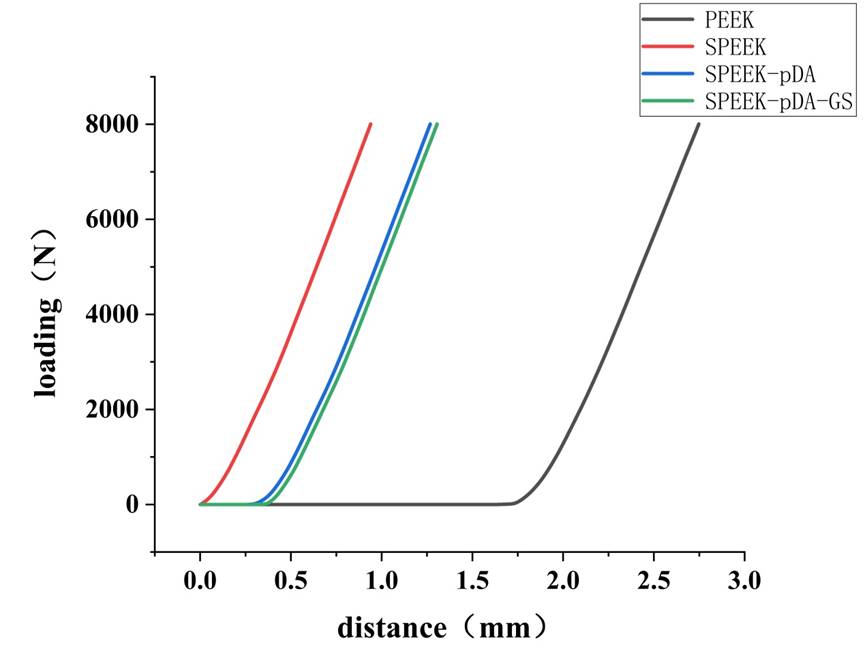


Fig. S2. Loading-distance curves of treated versus untreated PEEK samples, which confirm that the treatments do not affect the mechanical properties of PEEK samples.


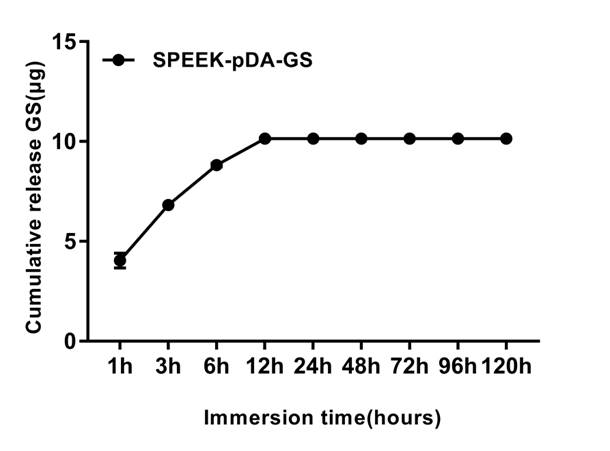


Fig. S3. The GS release behavior from SPEEK-pDA-GS in terms of cumulative release.


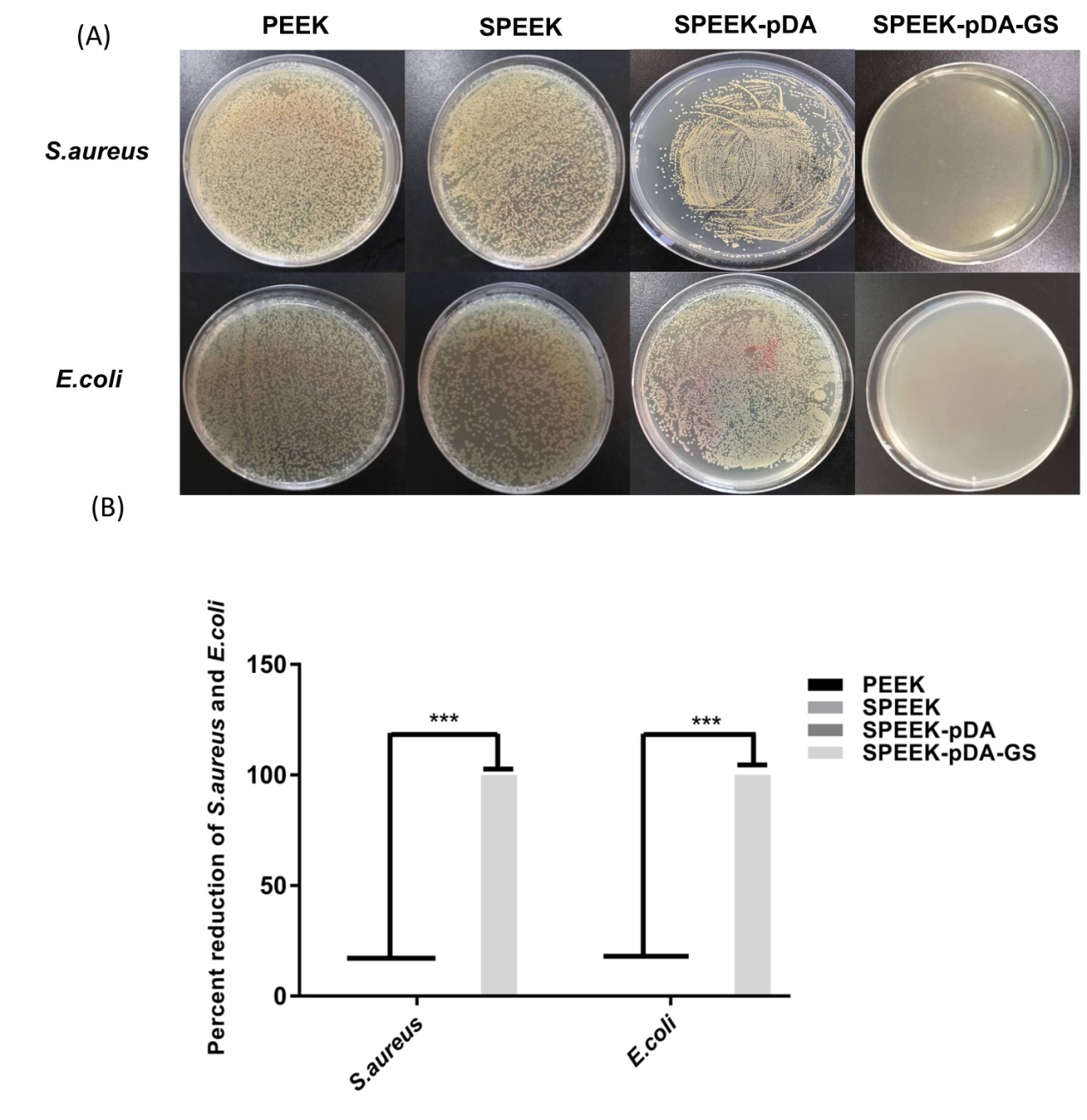


Fig. S4. Bacteria colonies (A) and the reduction percentages (B) of 10^7^cfu/mL *S. aureus* and *E. coli* co-cultured with different modified PEEK for 24 h and then seeded on agar after dissociation from the various modified PEEK samples. (n=3, *** P < 0.001).


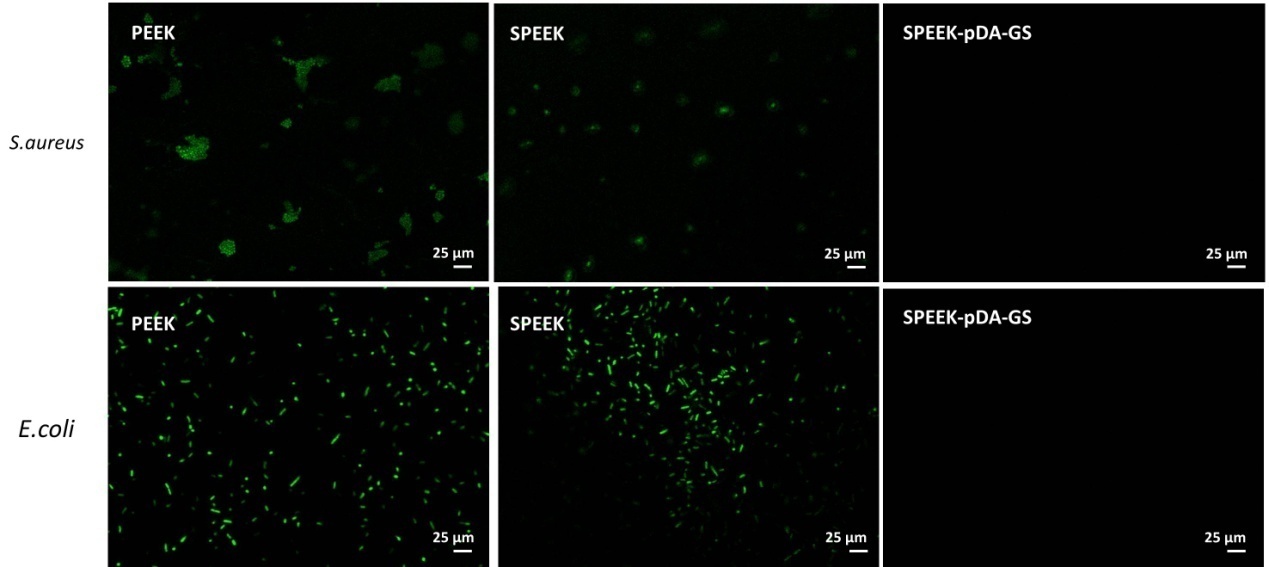


Fig. S5. Live/dead staining images of *S. aureus* and *E. coli* adhered on different PEEK samples after 24 h of incubation using SYTO9/PI.


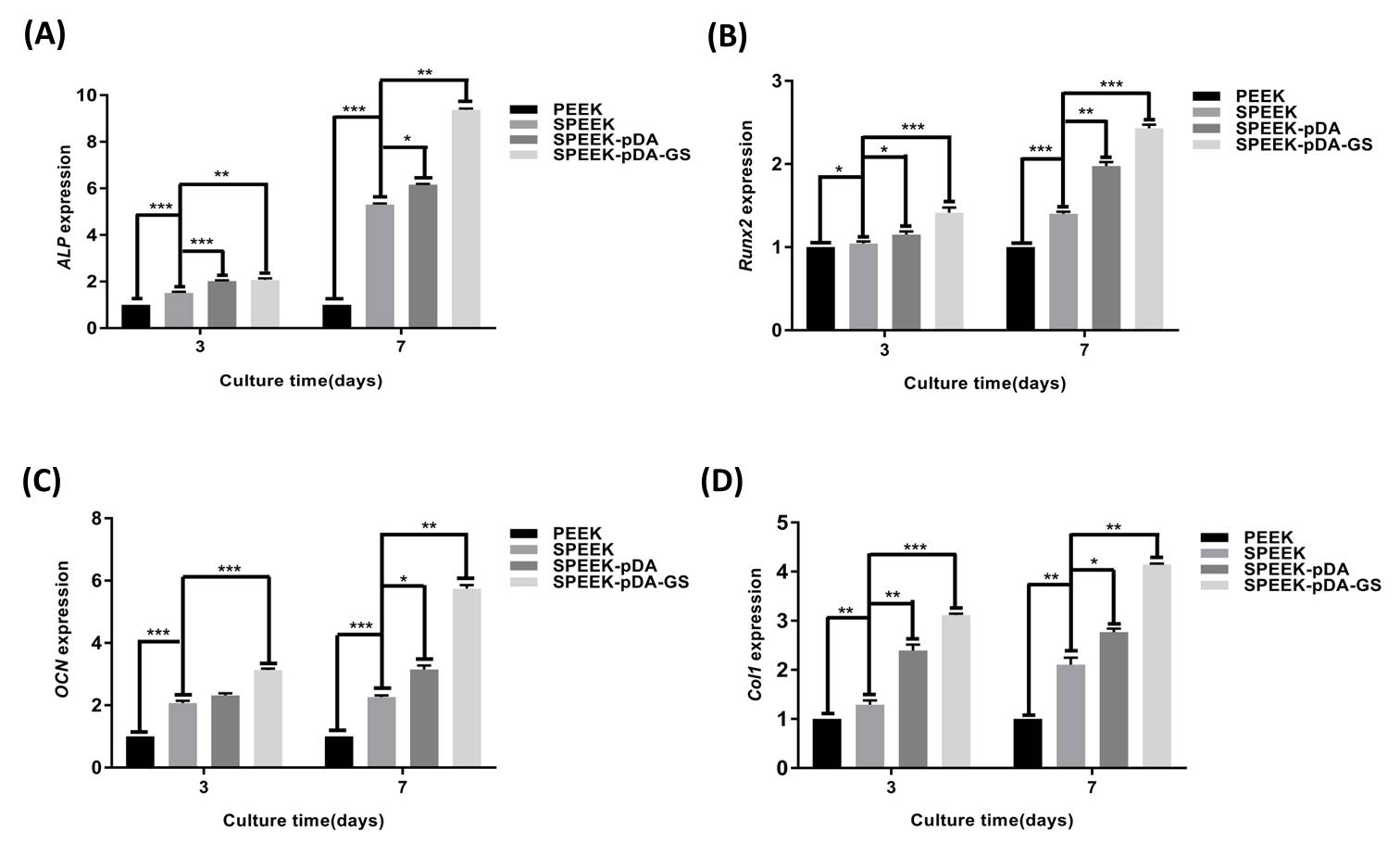


Fig. S6. Osteogenic marker expression in MC3T3-E1 cultured on PEEK, SPEEK, SPEEK-pDA and SPEEK-pDA-GS surfaces on day 3 and 7. RT-PCR analysis of osteo-related genes encoding ALP, Runx 2, OCN, and Col-I. (n=3, * p < 0.05, **p < 0.01, and ***p < 0.001).


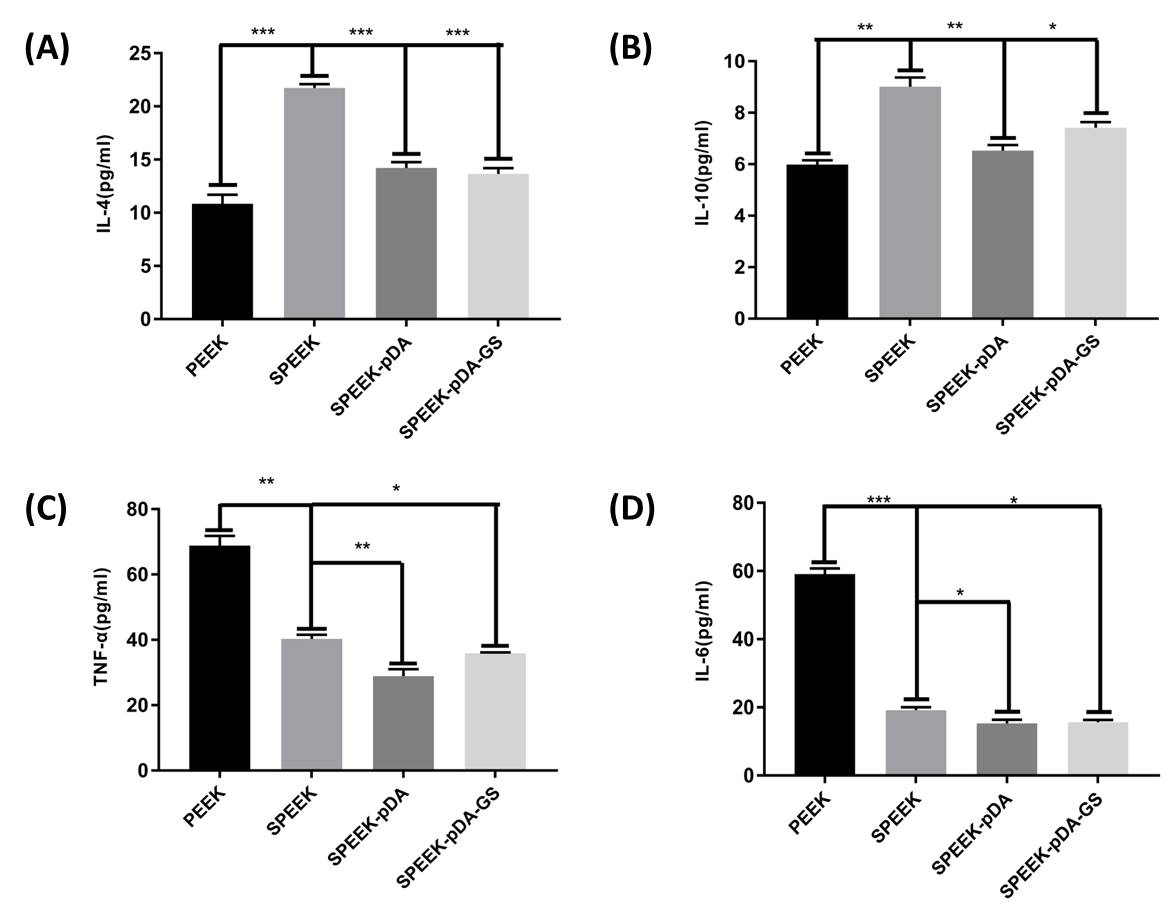


Fig. S7. ELISA results of the expression of IL-4, IL-10, TNF-ɑ and IL-6 of in RAW264.7 cultured on PEEK, SPEEK, SPEEK-pDA and SPEEK-pDA-GS for 3 days, respectively. (n=3, * p < 0.05, **p < 0.01, and ***p < 0.001).


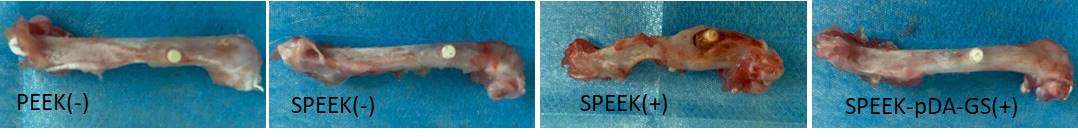


Fig. S8. Representative image of the harvested distal femurs, the surgical area and the placement of the implant in PEEK (-), SPEEK (-), SPEEK (+), and SPEEK–pDA–GS (+) groups, respectively.


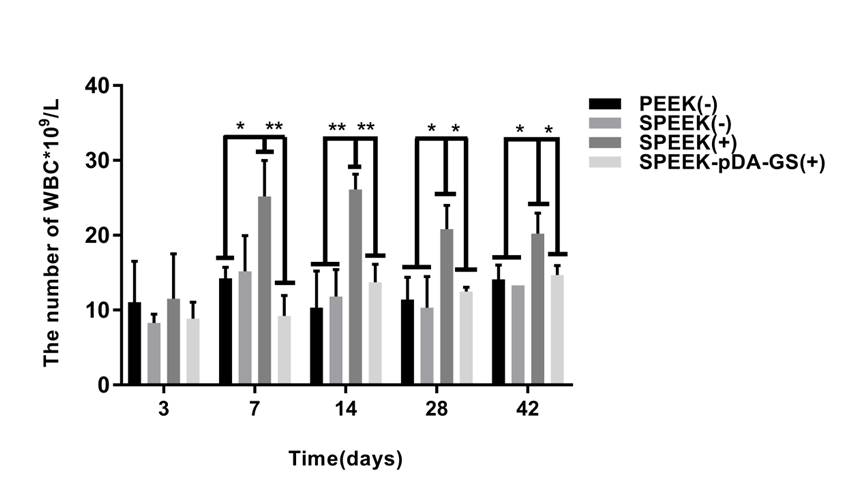


Fig. S9. Trend chart of white blood cell count in rats after operation with PEEK (-), SPEEK (-), SPEEK (+), and SPEEK–pDA–GS (+) groups, respectively.(n=3, * p < 0.05, **p < 0.01, and ***p < 0.001).


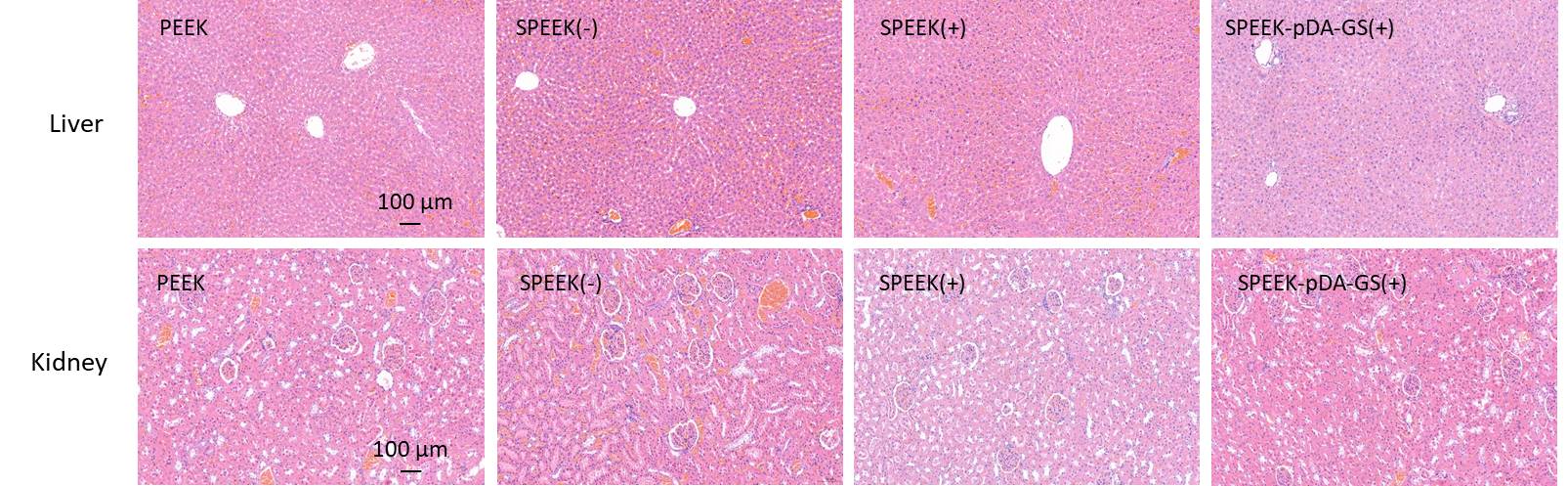


Fig. S10. Histological analysis using HE staining of liver and kidney of the experimental rats in PEEK (-), SPEEK (-), SPEEK (+), and SPEEK–pDA–GS (+) groups, respectively.
